# *GCKR* and *ADIPOQ* gene polymorphisms in women with gestational diabetes mellitus

**DOI:** 10.1007/s00592-023-02165-1

**Published:** 2023-07-31

**Authors:** Manning Zhu, Yaer Lv, Yanqing Peng, Yingnan Wu, Yanan Feng, Tianshuang Jia, Songcheng Xu, Songxue Li, Wei Wang, Jiawei Tian, Litao Sun

**Affiliations:** 1 Cancer Center, Department of Ultrasound Medicine, Zhejiang Provincial People′s Hospital(Affiliated People′s Hospital), Hangzhou Medical College, Hangzhou, Zhejiang China; 2 Department of Ultrasound, The Second Affiliated Hospital of Harbin Medical University, Harbin, Heilongjiang Province China

**Keywords:** Gestational diabetes mellitus, *GCKR*, *ADIPOQ*, SNPs

## Abstract

**Aims:**

To investigate the associations of *GCKR* and *ADIPOQ* variants with the risk of gestational diabetes mellitus (GDM) in Chinese women.

**Methods:**

*GCKR* rs1260326, *ADIPOQ* rs266729, and rs1501299 were selected and genotyped in 519 GDM patients and 498 controls. Candidate SNPs were genotyped using multiplex polymerase chain reaction (PCR) combined with next-generation sequencing methods, and the association of these SNPs with GDM was analyzed.

**Results:**

We found that *GCKR* rs1260326 was significantly associated with an increased risk of GDM in the allele model, the codominant model (CC vs. TT), the dominant model, the recessive model, and the genotypic model distributions (*p* = 0.0029, *p* = 0.0022, *p* = 0.0402, *p* = 0.0038, and *p* = 0.0028, respectively). The rs1260326 polymorphism was shown to be associated with 1 h-OGTT level and gravidity in GDM patients (CC vs. TT: *p* = 0.0475 and *p* = 0.0220, respectively). Diastolic blood pressure (DBP) was significantly higher in the GDM patients with the rs266729 GG genotype compared to those with the CC or CG genotype (*p* = 0.0444 and *p* = 0.0339, respectively). The DBP of the GDM patients with the rs1501299 GT genotype was lower than that of those with the GG genotype (*p* = 0.0197). There was a weak linkage disequilibrium value between the *GCKR* and *ADIPOQ* SNPs.

**Conclusions:**

The genes *GCKR* and *ADIPOQ* may be involved in the pathophysiology of GDM.

**Supplementary Information:**

The online version contains supplementary material available at 10.1007/s00592-023-02165-1.

## Introduction

Gestational diabetes mellitus (GDM) is defined as glucose intolerance with onset or first recognition during pregnancy, which is a common complication in pregnant women. GDM brings negative effects for pregnant women, fetuses, neonates, and children in the growth and development period, such as hypertensive disease in pregnancy, placental abruption, abortion, fetal macrosomia, fetal malformations, neonatal asphyxia, and cognitive dysfunction in children in the growth and development period. The risks of type 2 diabetes mellitus (T2DM) and cardiovascular disease in pregnant women and their offspring are increased [1]. GDM is caused by a number of factors, the most important of which are insulin resistance and pancreatic islet β cell dysfunction. The incidence of GDM in pregnant women with a family history of diabetes was significantly higher, which revealed that genes played an important role in the pathogenesis of GDM. In humans, single nucleotide polymorphisms (SNPs) are the most prevalent kind of genetic variation. They relate to single nucleotide modifications at certain genomic locations. SNPs can be utilized to predict GDM risk. A large amount of previous evidence has shown that some genetic variations were associated with GDM [2–4].

The *GCKR* gene is located on chromosome 2p23.3 and contains 19 exons. The encoded glucokinase regulatory protein (GKRP) regulates glycolysis by inhibiting the enzyme activity of the glycolytic enzyme glucokinase (GCK) at low glucose concentrations [5]. GCK is responsible for glucose phosphorylation in the glycolysis pathway. Therefore, it is crucial for preserving blood glucose homeostasis [6]. The overexpression of *GCKR* in the liver causes an increase in GCK activity, which lowers glucose levels while raising triglyceride levels. The *ADIPOQ* gene is located on chromosome 3q27 and contains 3 exons and 2 introns. The encoded adiponectin increases the phosphorylation of AMP-activated protein kinase (AMPK) and p38 mitogen-activated protein kinase (p38 MAPK), as well as the peroxisome proliferator-activated receptor (PPAR) ligand activity after binding with its receptors, which plays a role in the down-regulation of key gluconeogenesis enzymes, the promotion of fatty acid oxidation, and the increase of glucose uptake [7, 8].

GDM is the prophase of T2DM to some extent, and they have similar pathophysiological changes and genetic characteristics. At present, the association studies of SNPs and GDM genetic susceptibility are mainly based on the association studies of T2DM genetic susceptibility. Tracing the genetic origin of GDM may help clarify the pathogenesis of the disease. Therefore, our study explored the correlations between *GCKR* and *ADIPOQ* gene polymorphisms and GDM in Chinese women so as to provide a new basis and direction for the clinical treatment of GDM in the future.

## Materials and methods

### Study subjects

The subjects were continuously recruited from the same center (Department of Obstetrics and Gynecology, the Second Affiliated Hospital of Harbin Medical University, Harbin, Heilongjiang Province, China) from December 2016 to December 2018. There were 1157 pregnant women in the research, including 560 pregnant women with GDM and 597 pregnant women with a normal oral glucose tolerance test (OGTT). The diagnosis of GDM was based on a 75-g OGTT at 24–28 weeks’ gestation, according to the 2015 International Association of Diabetes and Pregnancy Study Groups (IADPSG) criteria [9]. The diagnosis of GDM was made when one of the following plasma glucose values in the OGTT was met or exceeded: fasting plasma glucose 92 mg/dL (5.1 mmol/L), 1-h plasma glucose 180 mg/dL (10.0 mmol/L), and 2-h plasma glucose 153 mg/dL (8.5 mmol/L) [9]. The exclusion criteria were pre-gestational diabetes mellitus (n = 20), multiple pregnancies (n = 17), hypertension (n = 21), ethnic minorities (n = 14), liver and renal disfunction (n = 16), complicated with systemic metabolic diseases such as thyroid dysfunction (n = 15), systemic lupus erythematosus (n = 6), rheumatoid disease (n = 5), and other diseases that may cause abnormal blood glucose during pregnancy (n = 7). During the genotyping process, cases that could not be completely genotyped were excluded (n = 19). After exclusion, 1017 pregnant women (519 GDM patients and 498 controls) were recruited (Supplementary Figure S1).

All participants agreed with the ethics of the study and signed informed consent, which was approved by the ethics committee of the Second Affiliated Hospital of Harbin Medical University.

### Selection of SNPs

Recent genome-wide association studies (GWAS) identified *GCKR* and *ADIPOQ* as being associated with T2DM and the metabolic syndrome [10–12]. Blood glucose control and lipid balance were significantly influenced by *GCKR* and *ADIPOQ* gene polymorphisms. However, the influence of these genetic variants on GDM in the general population is unclear and controversial [13, 14]. Based on the results of T2DM GWAS [15], minor allele frequency (MAF) > 0.15 in the Chinese population, and tracking the relevant literature [16, 17], we finally selected three candidate SNPs (*GCKR* rs1260326, *ADIPOQ* rs266729, and rs1501299) that might be associated with GDM. *GCKR* rs1260326 (T > C) is a missense polymorphism that causes a Leu to Pro substitution (P446L). rs266729 (C > G) is located in the promoter region of the *ADIPOQ* gene, and rs1501299 (G > T) is located in intron 2 of the *ADIPOQ* gene.

### Extraction and genotyping of DNA

Each subject had a peripheral venous blood sample (4–5 ml) collected into a 2% EDTA-Na2 anticoagulant tube, which was then stored at -80 °C until DNA extraction. TIANamp Genomic DNA Kit from Tiangen Biotech, China, was used to extract genomic DNA from peripheral blood samples. Genotyping of the selected *GCKR* rs1260326, *ADIPOQ* rs266729, and rs1501299 was tested using multiplex PCR combined with next-generation sequencing methods by the Shanghai Bio Wing Applied Biotechnology Company (http://www.biowing.com.cn) [18]. Primer3 online software (version 0.4.0, http://frodo.wi.mit.edu/) was used to amplify primer sequences. The primers used for amplification are as follows: for rs1260326 forward 5′-CTATAGTGGAGCAGGTGAAAGAG-3′ and reverse 5′-TCATATTCAAAGAAAAGCAGTGGC-3′; for rs266729 forward 5′-GTTTTGGATGTCTTGTTGAAGTTG-3′ and reverse 5′-CTAGAAAGTTTAGGCTTGAAGTGG-3′; for rs1501299 forward 5′-GTTATAGAGGCACCATCTACACTC-3′ and reverse 5′-GAGATCCAGGTAAGAATGTTTCTG-3′. TIANgel Midi Purification Kit (Tiangen Biotech, China) was utilized for purifying the PCR products after PCR amplification was performed. The purified PCR products were performed by Illumina HiSeq XTen platform with paired-end sequencing (2 × 150 bp) according to the manufacturer’s instructions. The Burrows-Wheeler Aligner (BWA, v0.7.12) was used to align the sequences to the human reference genome, and Samtools (v0.1.19) was used for SNP calling and genotyping [19]. Some samples were randomly selected for blind DNA replication for quality control in genotyping.

### Statistics

The genotypic distributions of each SNP in the GDM patients and controls were tested for departure from Hardy–Weinberg equilibrium (HWE) using an exact test. Kolmogorov–Smirnov test was used to analyze the distribution of continuous patient demographic and clinical characteristic data. Normally distributed continuous data were compared using analysis of variance (ANOVA), and non-normally distributed continuous data were compared using rank sum test. Categorical, normally distributed continuous, and non-normally distributed data are shown as number (n) and percentage (%), mean ± standard deviation (SD), and median and interquartile range (IQR), respectively. The 95% confidence interval (CI) and odds ratio (OR) were calculated through multiple logistic regression analysis to evaluate the potential association between *GCKR* and *ADIPOQ* gene polymorphisms and GDM. Adjusted ORs were computed with adjustment for confounding factors that included maternal age, gestational age, BMI before pregnancy, BMI at enrollment, systolic blood pressure (SBP), diastolic blood pressure (DBP), birth weight, urea, prothrombin time (PT), activated partial thromboplastin time (APTT), and gravidity, then calculated using 10,000 permutations for each model to correct the multiple test. The statistical analyses were performed using IBM SPSS 24.0 Statistics and R 4.0.0. The SHEsis software was used to assess linkage disequilibrium between pair of SNPs [20].

## Results

### Quality control and SNP genotype

All of the tested SNPs were in agreement with the Hardy–Weinberg Equilibrium (HWE) in the GDM patients and controls of this study (*p* > 0.05), as shown in Supplementary Table S1. In addition, quality control was set up for the genotypes of several samples. The genotype calling rate in 115 quality control samples was 98.70%, which fully improved the reliability of the follow-up research results.

### Clinical characteristics of the study population

The clinical characteristics of the GDM patients and controls were shown in Table 1. The maternal age, gestational age, BMI before pregnancy, BMI at enrollment, SBP, DBP, birth weight, urea, PT, APTT, and gravidity of the GDM patients and controls were significantly different (*p* < 0.05).

**Table 1:** Clinical characteristics of the GDM patients and controls

Variables	Controls	Patients	*p*
Numbers (n)	498	519	
Maternal age (year)	29.00 (27.00–32.00)	31.00 (28.00–34.00)	** < .0001**
Age < 35	422 (84.7)	392 (75.5)	**0.0002**
Age ≥ 35	76 (15.3)	127 (24.5)	
Gestational age (week)	39.00 (39.00–40.00)	39.00 (38.00–40.00)	** < .0001**
BMI before pregnancy (kg/m^2^)	20.64 (19.00–22.66)	22.43 (20.20–24.61)	** < .0001**
Underweight, 18.5	91 (18.3)	54 (11.0)	** < .0001**
Normal weight, 18.5–23.9	339 (68.2)	283 (57.9)	
Overweight, 24.0–27.9	54 (10.9)	106 (21.7)	
Obese, ≥ 28	13 (2.6)	46 (9.4)	
BMI at enrollment (kg/m^2^)	26.95 (24.80–28.91)	27.93 (25.56–30.41)	** < .0001**
SBP (mmHg)	116.00 (110.00–122.00)	120.00 (113.00–126.00)	** < .0001**
DBP (mmHg)	78.00 (74.00–84.00)	81.00 (75.00–87.00)	** < .0001**
Birth weight (g)	3350.00 (3000.00–3700.00)	3450.00 (3150.00–3750.00)	**0.0055**
Urea (mmol/L)	3.28 (2.71–3.89)	3.42 (2.85–4.12)	**0.0031**
Creatinine (umol/L)	48.00 (43.00–54.00)	48.00 (42.00–56.00)	0.8988
PT (Sec)	9.80 (9.50–10.10)	9.90 (9.60–10.20)	**0.0216**
PTA (%)	108.00 (101.00–116.00)	107.00 (100.00–115.00)	0.1272
APTT (Sec)	29.60 (28.20–31.10)	29.10 (27.60–31.00)	**0.0087**
TT (Sec)	13.00 (12.40–13.80)	13.00 (12.50–13.70)	0.5616
Gravidity (n/%)	1.00 (1.00–2.00)	2.00 (1.00–2.00)	**0.0037**
1	271 (54.4)	236 (46.4)	**0.0149**
2	137 (27.5)	148 (29.1)	
≥ 3	90 (18.1)	125 (24.6)	

*p* < 0.05 was considered as statistically significant (bold)

*BMI* body mass index, *SBP* systolic blood pressure, *DBP* diastolic blood pressure, *PT* prothrombin time, *PTA* prothrombin activity, *APTT* activated partial thromboplastin time, *TT* thrombin time, *p* *p* value

### Genotype and allele association analysis

The genotypes and alleles frequencies of *GCKR* rs1260326, *ADIPOQ* rs266729, and rs1501299 in the GDM patients and controls were further analyzed, as shown in Table 2. The frequency and distribution of rs1260326 genotypes and alleles were significantly different between the GDM patients and controls (*p* = 0.0208 and *p* = 0.0080, respectively). For rs1260326, after adjusting for maternal age, gestational age, BMI before pregnancy, BMI at enrollment, SBP, DBP, birth weight, urea, PT, APTT, and gravidity, the allele model, the codominant model (CC vs. TT), the dominant model, the recessive model, and the genotypic model distributions were different between the GDM patients and controls (*p* = 0.0029, *p* = 0.0022, *p* = 0.0402, *p* = 0.0038, and *p* = 0.0028, respectively). After 10,000 permutations, the results were still statistically significant (*p* < 0.05, all). The genotypes and alleles frequencies of rs266729 were not significantly different in the GDM patients compared with controls (*p* > 0.05, all), and there was no significant difference between the GDM patients and controls under any model (*p* > 0.05, all). The frequency and distribution of rs1501299 alleles were significantly different between the GDM patients group and the control group (*p* = 0.0320). An analysis of the study between the two groups showed a statistically significant difference under the codominant model (TT vs. GG) of the rs1501299 polymorphism (*p* = 0.0457). However, the significance disappeared after adjusting for confounding factors (*p* > 0.05).

**Table 2:** Association of SNPs in *GCKR* and *ADIPOQ* with GDM risk

SNP	Controls	Patients	*p*^a^	Model		*p*^b^	OR (95% CI)	*p*^c^	OR (95% CI)	*p*^d^
rs1260326										
TT	164(33.1)	142(28.1)		Allele	C vs. T	**0.0081**	1.269(1.064–1.514)	**0.0029**	1.381(1.117–1.709)	**0.0045**
TC	245(49.5)	241(47.7)		Codominant	CC vs. TT	**0.0065**	1.638(1.148–2.339)	**0.0022**	1.984(1.279–3.077)	**0.0037**
CC	86(17.4)	122(24.2)	**0.0208**		TC vs. TT	0.3832	1.136(0.853–1.513)	0.3180	1.192(0.844–1.684)	0.3154
				Dominant	CC + TC vs. TT	0.0533	1.303(0.996–1.705)	**0.0402**	1.406(1.015–1.948)	**0.0461**
T	573(57.9)	525(52.0)		Recessive	CC vs. TC + TT	**0.0140**	1.472(1.081–2.004)	**0.0038**	1.735(1.195–2.519)	**0.0016**
C	417(42.1)	485(48.0)	**0.0080**	Overdominant	TC vs. CC + TT	0.8318	0.974(0.761–1.245)	0.6463	0.933(0.693–1.255)	0.6348
				Genotypic	CC vs. TC vs. TT	**0.0077**	1.270(1.065–1.514)	**0.0028**	1.385(1.119–1.714)	**0.0019**
rs266729										
CC	254(51.4)	259(52.1)		Allele	G vs. C	0.9735	0.997(0.819–1.213)	0.5976	0.938(0.739–1.190)	0.6073
CG	206(41.7)	201(40.4)		Codominant	GG vs. CC	0.7975	1.067(0.649–1.754)	0.5501	0.827(0.444–1.541)	0.5544
GG	34(6.88)	37(7.44)	0.8923		CG vs. CC	0.7400	0.957(0.738–1.241)	0.7409	0.948(0.692–1.300)	0.7482
				Dominant	GG + CG vs. CC	0.7257	1.045(0.817–1.336)	0.9903	0.998(0.741–1.345)	0.9627
C	714(72.3)	719(72.3)		Recessive	GG vs. CG + CC	0.8503	1.048(0.646–1.698)	0.6109	0.860(0.480–1.540)	0.6268
G	274(27.7)	275(27.7)	0.9735	Overdominant	CG vs. GG + CC	0.7968	1.033(0.806–1.325)	0.8018	1.039(0.769–1.405)	0.8241
				Genotypic	GG vs. CG vs. CC	0.7189	1.037(0.851–1.264)	0.8263	0.973(0.766–1.237)	0.8103
rs1501299										
GG	254(51.4)	284(57.0)		Allele	T vs. G	0.0324	0.804(0.658–0.982)	0.0983	0.816(0.642–1.038)	0.1124
GT	198(40.1)	186(37.3)		Codominant	TT vs. GG	**0.0457**	0.596(0.359–0.990)	0.1265	0.624(0.340–1.143)	0.1350
TT	42(8.50)	28(5.62)	0.0893		GT vs. GG	0.1930	0.840(0.646–1.092)	0.2860	0.841(0.611–1.157)	0.2883
				Dominant	TT + GT vs. GG	0.2355	0.861(0.673–1.102)	0.3310	0.862(0.640–1.163)	0.2889
G	706(71.5)	754(75.7)		Recessive	TT vs. GT + GG	0.0576	0.619(0.377–1.016)	0.1583	0.654(0.363–1.180)	0.1518
T	282(28.5)	242(24.3)	**0.0320**	Overdominant	GT vs. TT + GG	0.8256	0.972(0.757–1.249)	0.8142	0.964(0.711–1.308)	0.8317
				Genotypic	TT vs. GT vs. GG	0.0834	0.839(0.688–1.023)	0.1737	0.847(0.667–1.076)	0.1695

*p*, *p* value; *p* < 0.05 was considered as statistically significant (bold)

^a^Comparison between GDM and controls

^b^Calculated using multivariate logistic regression analysis

^c^Adjusted by maternal age, gestational age, BMI before pregnancy, BMI at enrollment, SBP, DBP, birth weight, urea, PT, APTT, and gravidity

^d^Adjusted by maternal age, gestational age, BMI before pregnancy, BMI at enrollment, SBP, DBP, birth weight, urea, PT, APTT, and gravidity, then calculated using 10,000 permutations for each model to correct the multiple test

**Fig. 1:**
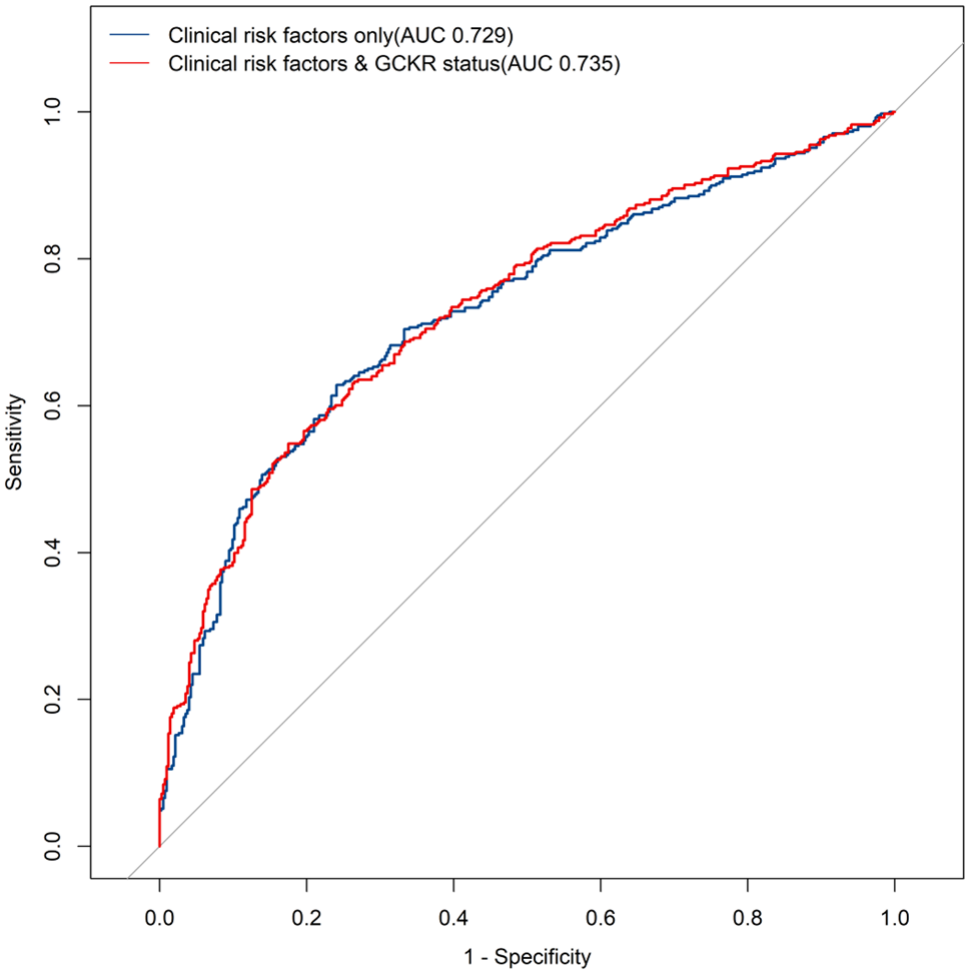
ROC curves for prediction of GDM. Clinical risk factors include maternal age, gestational age, BMI before pregnancy, BMI at enrollment, SBP, DBP, birth weight, urea, PT, APTT, and gravidity

In ROC analysis, the AUC for clinical risk factors (maternal age, gestational age, BMI before pregnancy, BMI at enrollment, SBP, DBP, birth weight, urea, PT, APTT, and gravidity) was 0.729 (95% CI 0.694, 0.763) (Fig. 1). Addition of rs1260326 status (TC or CC) increased the predictive value of the model, giving an AUC of 0.735 (95% CI 0.701, 0.769).

We further investigated the association between *GCKR* rs1260326, *ADIPOQ* rs266729, and rs1501299 polymorphisms and clinical information in patients with GDM. Stratified analysis was performed to analyze the association between the genotypes of the three SNPs and maternal age, gestational age, BMI before pregnancy, BMI at enrollment, SBP, DBP, birth weight, urea, PT, APTT, gravidity, HbA1c, FBG, 1 h-OGTT, 2 h-OGTT. As shown in Tables 3, 4, and 5, a significant association was demonstrated between rs1260326 polymorphism and 1 h-OGTT level and gravidity in patients with GDM (CC vs. TT: *p* = 0.0475 and *p* = 0.0220, respectively). We observed that DBP was significantly higher in the GDM patients with the rs266729 GG genotype compared to those with the CC or CG genotype (*p* = 0.0444 and *p* = 0.0339, respectively). The DBP of the GDM patients with the rs1501299 GT genotype was lower than that of those with the GG genotype (*p* = 0.0197).

**Table 3:** Analysis of *GCKR* rs1260326 genotype in GDM patients by clinical features

Variables	TT	TC	CC	*p*	CC vs. TT	TC vs. TT	CC vs. TC
Maternal age (year)	30.00 (28.00–35.00)	31.00 (29.00–34.00)	31.00 (28.00–35.00)	0.8206	0.7879	0.9758	0.9005
Gestational age (week)	39.00 (38.00–40.00)	39.00 (38.00–40.00)	39.00 (38.00–40.00)	0.5550	0.9562	0.5502	0.7863
BMI before pregnancy (kg/m^2^)	22.43 (20.20–24.22)	22.31 (20.20–24.50)	22.86 (20.06–25.34)	0.7131	0.8789	0.9403	0.6986
BMI at enrollment (kg/m^2^)	28.28 (26.17–30.41)	27.82 (25.44–30.22)	28.28 (25.34–30.44)	0.6514	0.8180	0.6201	0.9919
SBP (mmHg)	120.00 (114.00–126.00)	120.00 (112.00–125.00)	120.00 (113.50–125.50)	0.5453	0.9997	0.6585	0.6125
DBP (mmHg)	81.00 (76.00–88.00)	80.00 (75.00–85.50)	80.50 (75.00–87.00)	0.7112	0.8606	0.6866	0.9847
Birth weight (g)	3400.00 (3200.00–3700.00)	3450.00 (3200.00–3800.00)	3450.00 (3100.00–3750.00)	0.5840	0.8904	0.5489	0.8941
Urea (mmol/L)	3.38 (2.78–4.01)	3.43 (2.85–4.15)	3.50 (3.00–4.11)	0.7508	0.7530	0.8186	0.9797
PT (s)	9.90 (9.60–10.20)	9.90 (9.60–10.30)	9.80 (9.50–10.10)	0.7881	0.8183	0.9948	0.8073
APTT (s)	28.60 (27.30–30.90)	29.20 (27.70–31.10)	29.20 (27.90–31.00)	0.2568	0.3666	0.2819	0.9927
Gravidity (n/%)	1.00 (1.00–2.00)	2.00 (1.00–3.00)	2.00 (1.00–3.00)	**0.0181**	**0.0220**	0.0561	0.7230
HbA1c (%)	5.60 (5.30–5.80)	5.50 (5.30–5.80)	5.60 (5.40–5.80)	0.4588	0.9937	0.5740	0.5434
FBG(mmol/L)	5.27 (5.10–5.48)	5.27 (5.03–5.50)	5.21 (4.94–5.58)	0.8189	0.9875	0.8898	0.8400
1 h-OGTT(mmol/L)	8.59 (7.38–10.16)	9.25 (7.95–10.40)	9.52 (7.86–10.72)	**0.0384**	**0.0475**	0.1213	0.6035
2 h-OGTT(mmol/L)	8.15 (7.02–9.24)	8.25 (7.10–9.17)	8.55 (7.18–9.18)	0.7188	0.7029	0.9734	0.8010

*p* < 0.05 was considered as statistically significant (bold)

*BMI* body mass index, *SBP* systolic blood pressure, *DBP* diastolic blood pressure, *PT* prothrombin time, *APTT* activated partial thromboplastin time, *p* *p* value

**Table 4:** Analysis of *ADIPOQ* rs266729 genotype in GDM patients by clinical features

Variables	CC	CG	GG	*p*	GG vs. CC	CG vs. CC	GG vs. CG
Maternal age (year)	31.00 (28.00–34.00)	30.00 (29.00–35.00)	31.00 (29.00–35.00)	0.5840	0.7485	0.6464	0.9681
Gestational age (week)	39.00 (38.00–40.00)	39.00 (38.00–40.00)	39.00 (39.00–40.00)	0.6532	0.7265	0.9393	0.6304
BMI before pregnancy (kg/m^2^)	22.48 (20.26–24.78)	22.36 (20.20–24.49)	22.50 (20.00–23.88)	0.8861	0.8870	0.9985	0.8804
BMI at enrollment (kg/m^2^)	27.92 (25.34–30.41)	27.99 (26.03–30.44)	28.52 (25.28–29.73)	0.8257	1.0000	0.8131	0.9610
SBP (mmHg)	119.00 (113.00–125.00)	120.00 (113.00–126.00)	120.00 (112.00–130.00)	0.4690	0.4605	0.8962	0.5705
DBP (mmHg)	80.86 ± 8.92	80.62 ± 9.19	84.11 ± 10.45	0.0983	**0.0444**	0.7813	**0.0339**
Birth weight (g)	3400.00 (3150.00–3750.00)	3450.00 (3150.00–3750.00)	3450.00 (3300.00–3800.00)	0.6479	0.6447	0.9767	0.6654
Urea (mmol/L)	3.42 (2.84–4.20)	3.49 (2.87–4.08)	3.39 (2.85–3.87)	0.9209	0.9860	0.9173	0.9931
PT (s)	9.90 (9.60–10.20)	9.90 (9.60–10.20)	10.00 (9.70–10.30)	0.4185	0.4114	0.9978	0.4135
APTT (s)	29.10 (27.70–31.00)	28.90 (27.40–30.90)	29.90 (28.40–31.10)	0.1999	0.3679	0.7042	0.1723
Gravidity (n/%)	2.00 (1.00–2.00)	2.00 (1.00–3.00)	2.00 (1.00–3.00)	0.2659	0.6000	0.2871	0.9929
HbA1c (%)	5.60 (5.40–5.80)	5.60 (5.30–5.80)	5.75 (5.40–5.90)	0.4720	0.4893	0.9840	0.4371
FBG(mmol/L)	5.22 (4.92–5.49)	5.30 (5.10–5.57)	5.23 (5.05–5.45)	0.3219	0.9326	0.3723	0.5494
1 h-OGTT(mmol/L)	9.42 (7.77–10.50)	8.85 (7.73–10.13)	9.45 (7.96–10.81)	0.1531	0.9430	0.1845	0.3836
2 h-OGTT(mmol/L)	8.20 (7.09–9.12)	8.28 (7.31–9.12)	8.91 (6.99–9.59)	0.4705	0.5115	0.9269	0.4648

*p* < 0.05 was considered as statistically significant (bold)

*BMI* body mass index, *SBP* systolic blood pressure, *DBP* diastolic blood pressure, *PT* prothrombin time, *APTT* activated partial thromboplastin time, *p* *p* value

**Table 5:** Analysis of *ADIPOQ* rs1501299 genotype in GDM patients by clinical features

Variables	GG	GT	TT	*p*	TT vs. GG	GT vs. GG	TT vs. GT
Maternal age (year)	31.00(29.00–35.00)	30.00(28.00–34.00)	30.00(28.00–33.00)	0.5585	0.7791	0.6046	0.9778
Gestational age (week)	39.00(38.00–40.00)	39.00(38.00–40.00)	39.50(38.00–40.00)	0.2691	0.3238	0.8523	0.2586
BMI before pregnancy (kg/m^2^)	22.43(20.20–24.77)	22.49(20.13–24.27)	22.04(19.28–24.61)	0.8348	0.8218	0.9710	0.8925
BMI at enrollment (kg/m^2^)	27.92(25.65–30.44)	27.97(25.53–29.86)	27.85(24.61–29.40)	0.6949	0.6643	0.9571	0.7830
SBP (mmHg)	120.00(113.00–126.00)	120.00(112.00–126.00)	119.00(110.00–125.00)	0.4436	0.3871	0.9583	0.5458
DBP (mmHg)	81.84 ± 9.27	79.79 ± 9.34	80.59 ± 7.64	0.0641	0.5038	**0.0197**	0.6720
Birth weight (g)	3450.00(3150.00–3750.00)	3425.00(3150.00–3775.00)	3400.00(3250.00–3625.00)	0.9025	0.8992	0.9973	0.9034
Urea (mmol/L)	3.41(2.78–4.12)	3.45(2.93–4.20)	3.49(3.02–3.68)	0.6518	0.9987	0.6440	0.8776
PT (s)	9.90(9.60–10.20)	9.90(9.60–10.20)	9.90(9.50–10.30)	0.8221	0.9699	0.8116	0.9987
APTT (s)	29.10(27.60–31.10)	29.20(27.90–30.90)	29.40(27.70–30.50)	0.9584	0.9609	0.9983	0.9528
Gravidity (n/%)	2.00(1.00–3.00)	2.00(1.00–2.00)	1.00(1.00–2.00)	0.5029	0.4859	0.9561	0.5405
HbA1c (%)	5.60(5.30–5.80)	5.60(5.30–5.80)	5.50(5.20–5.70)	0.5227	0.5396	0.9671	0.5084
FBG(mmol/L)	5.21(4.90–5.49)	5.29(5.11–5.51)	5.31(5.16–5.60)	0.0896	0.3042	0.1439	0.8533
1 h-OGTT(mmol/L)	9.23(7.90–10.37)	9.15(7.70–10.50)	8.04(7.66–9.74)	0.1753	0.1301	0.9825	0.2412
2 h-OGTT(mmol/L)	8.25(7.09–9.20)	8.52(7.30–9.27)	8.49(6.70–9.08)	0.5628	0.6370	0.8918	0.5694

*p* < 0.05 was considered as statistically significant (bold)

*BMI* body mass index, *SBP* systolic blood pressure, *DBP* diastolic blood pressure, *PT* prothrombin time, *APTT* activated partial thromboplastin time, *p* *p* value

### Linkage disequilibrium among the three SNPs

The linkage disequilibrium among the three SNPs (*GCKR* rs1260326, *ADIPOQ* rs266729, and rs1501299) were examined. It was found that rs1260326 vs. rs266729 (D’ = 0.056, r^2^ = 0.001), rs1260326 vs. rs1501299 (D’ = 0.013, r^2^ = 0.000), and rs266729 vs. rs1501299 (D’ = 0.030, r^2^ = 0.001) were in weak linkage disequilibrium (Supplementary Figure S2).

## Discussion

In the present study, we investigated the association between *GCKR* and *ADIPOQ* genetic variants and the risk of GDM in Chinese women. Through the analysis of the clinical data of GDM patients and controls, we found that the maternal age, gestational age, BMI before pregnancy, BMI at enrollment, SBP, DBP, birth weight, urea, PT, APTT, and gravidity of the GDM patients and controls were significantly different, especially the risk of GDM was higher in women with advanced maternal age (≥ 35 years old), which were consistent with the results of previous studies [21–23]. The rates of overweight, obesity, and higher gravidity (≥ 2) were significantly higher in the GDM patients compared with controls. The results also confirmed that GDM will increase the risk of hypertension in pregnancy and fetal macrosomia. Urea, PT, and APTT in patients with GDM were significantly different from those in controls. At present, studies have applied some biochemical indicators, such as APTT, to the prediction model of GDM [24].

To our knowledge, this is the first report that the *GCKR* rs1260326 polymorphism has been found to be associated with GDM. Our study showed that the C allele of rs1260326 increased the risk of GDM in Chinese women. The CC genotype of rs1260326 had a 1.984-fold increased risk of GDM in comparison to the TT genotype in the codominant model. *GCKR* rs1260326 is a missense polymorphism that causes leucine to proline substitution (P446L). With proline (encoded by the C allele of rs1260326) as opposed to leucine (encoded by the T allele of rs1260326) at position 446, GKRP responds more robustly to fructose-6-phosphate, resulting in more avid binding of glucokinase to GKRP, which leads to a decrease in glucokinase activity [25, 26]. Liu et al. [27] demonstrated that the C allele of rs1260326 was associated with greater insulin resistance. However, She et al. [28] reported the association between *GCKR* rs1260326 polymorphism and GDM in the Chinese Wuhan population, pointing out that there was no significant association between rs1260326 and GDM. There were few reports on the relationship between *GCKR* rs1260326 and GDM. Thus, the findings that *GCKR* rs1260326 may increase the risk of GDM need to be verified in the future.

Our results that the addition of genetic information to clinical risk factors modestly improved the prediction of GDM are consistent with several other researchers' findings [23, 29]. Clinical risk variables alone did not have as high a predictive value for GDM as those combined with the *GCKR* rs1260326 genotype. We identified genetic information associated with the risk of GDM, and the identified genetic polymorphisms could improve models’ predictive ability for GDM beyond classical risk factors and clinical markers. The findings may assist in early screening for and prevention of GDM and provide new insights into the mechanisms underlying GDM pathology.

A previous study showed that *GCKR* rs1260326 was significantly correlated with fasting blood glucose level [16]. Our subgroup analysis showed that GDM women with the rs1260326 CC genotype had a higher 1 h OGTT level compared to the TT genotype, which inspired us to speculate that there might be a timing effect on the association of *GCKR* SNPs with glycemic changes. Additional studies are warranted to validate the findings and clarify the underlying mechanism.

Our study found no evidence that the *ADIPOQ* rs266729 and rs1501299 were associated with GDM in Chinese women. Most studies on the correlation between *ADIPOQ* rs1501299 and GDM showed that there was no significant correlation between them [17, 30]. So far, only a study conducted by Shaat N et al. [31] suggested that the T allele of the *ADIPOQ* gene rs1501299 was associated with an increased risk of GDM in Scandinavia. rs1501299 is located in intron 2 of the *ADIPOQ* gene, which will be removed during mRNA post-transcriptional modification [32]. The correlation between *ADIPOQ* rs266729 and GDM was inconsistent in previous reports. In 2010, Liang Z et al. [33] found that the *ADIPOQ* gene rs266729 was related to GDM based on gene chip technology. In 2014, Beltcheva O et al. [30] reported that the G allele of the *ADIPOQ* gene rs266729 played a protective role in GDM to some extent. On the contrary, Pawlik A et al. [17] found that the G allele of the *ADIPOQ* gene rs266729 was associated with an increased risk of GDM in 2017. However, Silva et al. [34] believed that the *ADIPOQ* gene rs266729 had nothing to do with GDM, which was consistent with our research results. The reasons for these negative results remain unknown, but two possibilities should be considered. First, it may be because of genetic trait differences, as we know that genetic polymorphisms in human genes are distinct in different ethnicities, populations, and geographic regions. In addition, even though we might find a potential link between the disease-causing gene and the disease itself, GDM is a multi-factorial disease, and individual exposure to diverse environmental factors and genetic backgrounds may cause different results.

In addition, the subgroup analysis showed that, in the GDM patients, rs266729 and rs1501299 were associated with DBP, suggesting that the *ADIPOQ* gene polymorphisms genotypes may affect DBP in the GDM patients. An association between the T allele of rs1501299 and lower DBP has been reported in Amerindian subjects [35]. Significantly lower DBP in subjects with the mutated genotypes at rs1501299 were also reported in another study [36]. While the genetic background contributing specifically to the changes in DBP in GDM is still unknown, Studies have shown an inverse correlation between adiponectin and blood pressure [37, 38]. The exact role of these SNPs has yet to be determined, but it could be speculated that the *ADIPOQ* gene polymorphisms may influence adiponectin levels, which affect blood pressure by influencing endothelial function, regulating the renin-angiotensin system, and interacting with the sympathetic nervous system [39]. Therefore, it would be beneficial to conduct 24-h ambulatory blood pressure monitoring in further research and correlate the *ADIPOQ* gene polymorphisms with variations in blood pressure levels via adiponectin concentration.

So far as we know, this is the first study that analyzes the associations of the SNPs of *GCKR* rs1260326, *ADIPOQ* rs266729, and rs1501299 with GDM risk in the northeastern Han Chinese population and confirms that *GCKR* rs1260326 increased the risk of GDM. There were some deficiencies in this study. First of all, we did not detect the levels and activities of *GCKR* and *ADIPOQ* in the blood of the subjects, nor could we analyze the relationship between gene polymorphisms and their expression levels. Second, in the analysis of GDM genetic susceptibility, even after we adjusted for confounding variables such as maternal age, gestational age, BMI before pregnancy, BMI at enrollment, SBP, DBP, birth weight, urea, PT, APTT, and gravidity, these unmatched characteristics between groups may affect the results of the study. We also could not rule out the possibility that normal control pregnant women would develop GDM in subsequent pregnancies, which could result in grouping error and a reduction in the impact of genetic factors on the risk of GDM. Finally, our study population was limited to Han people in northeast China. People from different regions and ethnic backgrounds should be included, and replication of our findings in a broader population is warranted.

## Conclusions

In summary, the CC genotype of rs1260326 increased the risk of GDM in Chinese women. The addition of rs1260326 to clinical risk factors modestly improved the prediction of GDM. *GCKR* SNPs may have a timing effect on glycemic changes in GDM patients. Different *ADIPOQ* rs266729 or rs1501299 genotypes carried by GDM women can have different effects on DBP. In addition, we identified weak linkage disequilibrium between *GCKR* and *ADIPOQ* SNPs.

### Supplementary Information

Below is the link to the electronic supplementary material.

Supplementary file1 (DOCX 72 kb)

## Data Availability

SNP date is available in the Figshare database (https://figshare.com/), https://doi.org/10.6084/m9.figshare.23001491.v1.
