# Supplementary material for: GCKR and ADIPOQ gene polymorphisms in women with gestational diabetes mellitus
Source: Acta Diabetol. 2023 Jul 31;60(12):1709–18. doi: 10.1007/s00592-023-02165-1 (PMC10587232; doi:10.1007/s00592-023-02165-1)
Supplement: Supplementary file 1 — Supplementary file1 (DOCX 72 kb) [file 592_2023_2165_MOESM1_ESM.docx]

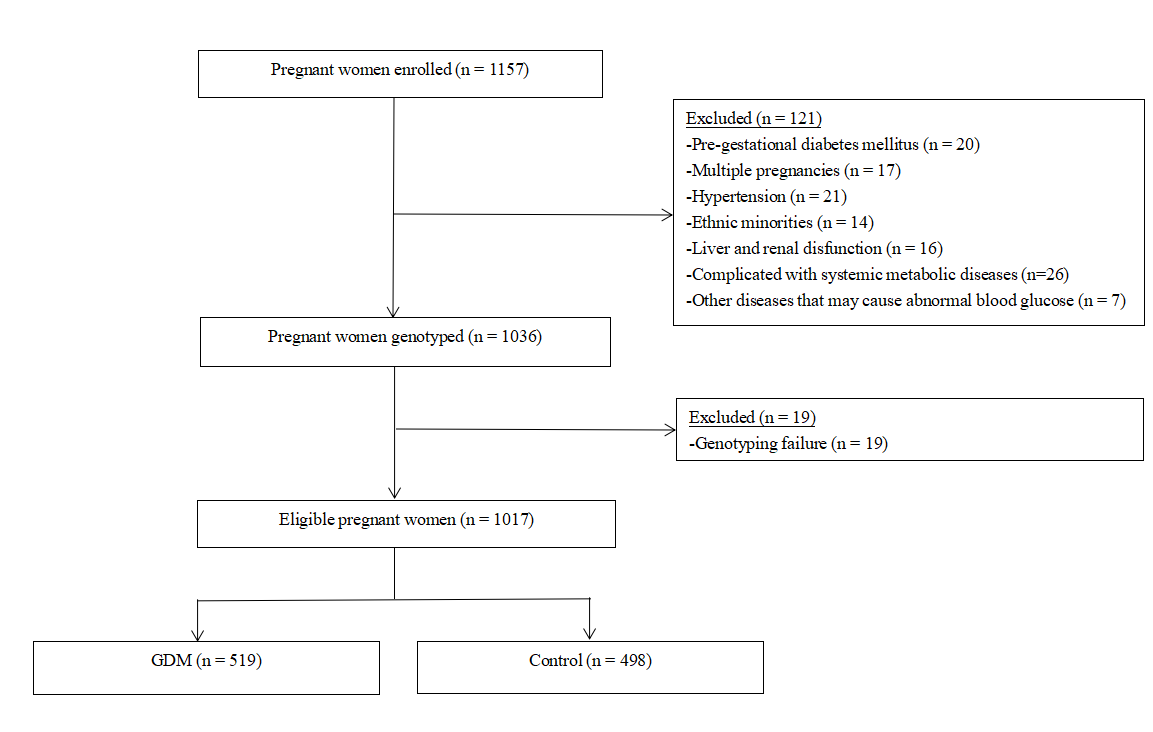


Supplementary Figure S1 The participant flowchart

Supplementary Table S1 The Hardy-Weinberg's expectation test of *GCKR* rs1260326, *ADIPOQ* rs266729, and rs1501299 in the GDM patients and controls

| **Single nucleotide polymorphism** | **Patients** | | **Controls** | |
| --- | --- | --- | --- | --- |
|  | **χ2** | ***p*** | **χ2** | ***p*** |
| rs1260326 | 0.980 | 0.322 | 0.113 | 0.737 |
| rs266729 | 0.054 | 0.816 | 0.804 | 0.370 |
| rs1501299 | 0.116 | 0.733 | 0.150 | 0.699 |

*p*, *p* value


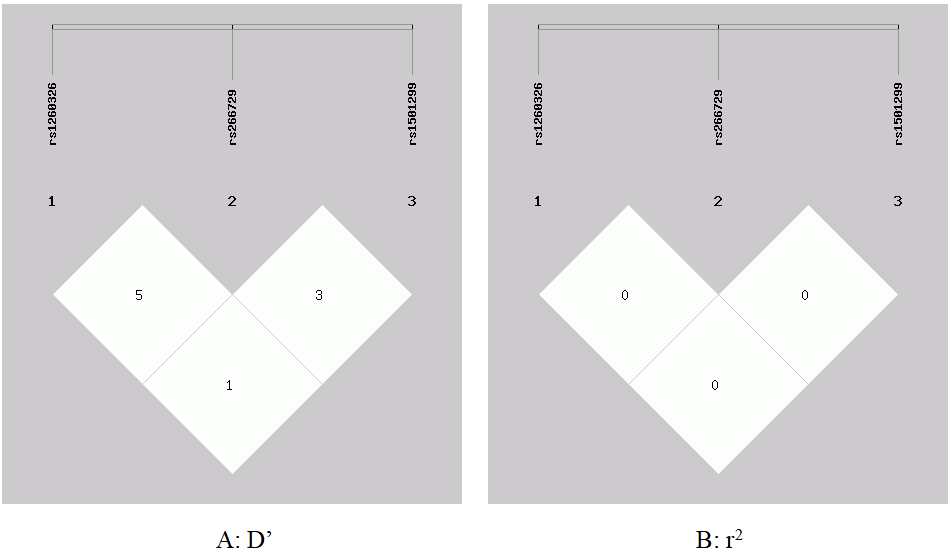


Supplementary Figure S2 Linkage disequilibrium analyses of rs1260326, rs266729, and rs1501299 SNPs in the GDM patients and controls
